# Transcription Factor Binding Profiles Reveal Cyclic Expression of Human Protein-coding Genes and Non-coding RNAs

**DOI:** 10.1371/journal.pcbi.1003132

**Published:** 2013-07-11

**Authors:** Chao Cheng, Matthew Ung, Gavin D. Grant, Michael L. Whitfield

**Affiliations:** 1 Department of Genetics, Geisel School of Medicine at Dartmouth, Hanover, New Hampshire, United States of America; 2 Institute for Quantitative Biomedical Sciences, Norris Cotton Cancer Center, Geisel School of Medicine at Dartmouth, Lebanon, New Hampshire, United States of America; EMBL-European Bioinformatics Institute & Wellcome Trust Sanger Institute, United Kingdom

## Abstract

Cell cycle is a complex and highly supervised process that must proceed with regulatory precision to achieve successful cellular division. Despite the wide application, microarray time course experiments have several limitations in identifying cell cycle genes. We thus propose a computational model to predict human cell cycle genes based on transcription factor (TF) binding and regulatory motif information in their promoters. We utilize ENCODE ChIP-seq data and motif information as predictors to discriminate cell cycle against non-cell cycle genes. Our results show that both the trans- TF features and the cis- motif features are predictive of cell cycle genes, and a combination of the two types of features can further improve prediction accuracy. We apply our model to a complete list of GENCODE promoters to predict novel cell cycle driving promoters for both protein-coding genes and non-coding RNAs such as lincRNAs. We find that a similar percentage of lincRNAs are cell cycle regulated as protein-coding genes, suggesting the importance of non-coding RNAs in cell cycle division. The model we propose here provides not only a practical tool for identifying novel cell cycle genes with high accuracy, but also new insights on cell cycle regulation by TFs and cis-regulatory elements.

## Author Summary

Cell cycle is a complex and highly supervised process that must proceed with regulatory precision to achieve successful cellular division. Microarray time course experiments have been successfully used to identify cell cycle regulated genes but with several limitations, e.g. less effective in identifying genes with low expression. We propose a computational approach to predict cell cycle genes based on TF binding data and motif information in their promoters. Specifically, we take advantage of ChIP-seq TF binding data generated by the ENCODE project and the TF binding motif information available from public databases. These data were processed and utilized as predictor for predicting cell cycle genes using the Random Forest method. Our results show that both the trans- TF features and the cis- motif features are predictive to cell cycle genes, and a combination of the two types features can further improve prediction accuracy. We apply our model to a complete list of GENCODE promoters to predict novel cell cycle driving promoters for both protein-coding genes and non-coding RNAs such as lincRNAs. We find that a similar percentage of lincRNAs are cell cycle regulated as protein-coding genes, suggesting the importance of non-coding RNAs in cell cycle division.

## Introduction

As one of the most important cellular processes, the cell division cycle is under precise regulation in all organisms. Mis-regulation of the cell cycle can lead to catastrophic cellular events, e.g. premature apoptosis or abnormal proliferation of cells, which are the causes of some human diseases such as cancer [1], [2]. Cell cycle regulation has been studied intensively, with focuses mainly on two aspects. First, cell cycle regulated genes have been identified systematically using microarrays to detect periodic expression of genes in cell cycle time course data [3], [4]. Second, the genes, particularly, the transcription factors (TFs) that modulate cell cycle have been investigated, e.g. identifying their genomic occupation using chromatin immunoprecipitation followed by microarray hybridization (ChIP-chip) or massively parallel sequencing (ChIP-seq) [5], [6]. These studies have provided many insights into cell cycle regulation during normal biological processes and in cancers.

Genome-wide gene expression during the cell cycle has been investigated using DNA microarrays in a wide range of organisms, including bacteria [7], yeast [3], [8]–[10], mouse [11], human [4], [12], [13] and Arabidopsis [14]. Microarray cell cycle time course data has been very successful at identifying a wide range of cell cycle-regulated genes. Despite its success, the microarray-based method has a few limitations. First, it is not effective for determining if a gene expressed at low levels is periodic due to low signal/noise ratios. Second, the synchronization procedure itself may change the expression pattern of some genes during the cell cycle, leading to false positive or false negative results. Third, limited by probe design, it is often difficult to distinguish expression patterns of different transcripts from the same gene. For example, for a gene with alternative promoter usage, it is possible that one isoform is cell cycle regulated while others are not. Consequently, the two isoforms may not be distinguished by a microarray based method if they share most of the exons. Moreover, previous microarray-based studies have focused on identification of cell cycle regulated protein-coding genes, while the non-coding RNAs have been largely overlooked. These issues can be overcome by measuring cell cycle gene expression using RNA-seq experiments, which unfortunately has not been performed.

The cell cycle is under precise gene regulation at different levels of expression [15], [16]. Particularly, at the transcriptional level it has been shown that a series of TFs act at different phases of the cell cycle and coordinate the sequential transcription of cell cycle genes [17]–[19]. The periodic expression pattern of cell cycle genes is encoded in cis in their promoters and can be manifested in trans by the TFs that bind to them. Namely, we would expect cell cycle genes to be bound by cell cycle regulating TFs. In this work, we raise and verify the hypothesis that cell cycle genes can be predicted by their genomic features (the motif occurrence in their promoters) and TF binding features (binding affinity of TFs). Recently, the ChIP-seq genomic binding data for a large number of human TFs have been published. In particular, the ENCODE (the Encyclopedia of DNA Elements) project has published binding profiles for more than 120 human TFs in different cell lines and more ChIP-seq data are being produced [20]. Motivated by these data, we aim to construct a model that integrates microarray cell cycle expression data with ChIP-seq TF binding data to predict new cell cycle genes and to understand the function of TFs in cell cycle regulation.

**Figure 1:**
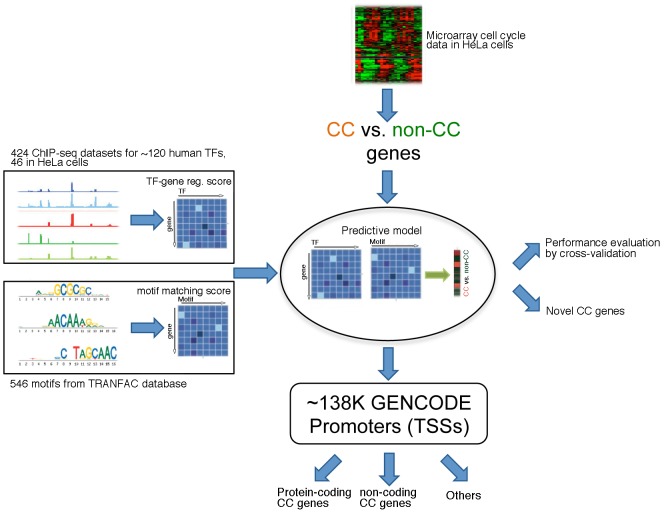
Schematic diagram of our analysis for predicting human cell cycle genes. The predictive model integrates three types of data from microarray, ChIP-seq experiments and computational TF binding motif analysis.

In this article, we present a computational method that predicts human cell cycle genes based on genomic and TF-binding features of genes. The model uses a supervised machine learning approach to integrate microarray cell cycle data, ChIP-seq TF binding data and motif information from sequence analysis (Figure 1). We first apply the model to all human RefSeq genes, which are well annotated with high accuracy. We validate the effectiveness of the model for cell cycle gene prediction by cross-validation, and we explore the relative importance of different predictors in the model. We then apply the model to the GENCODE TSS annotations, which provide a more comprehensive list of human promoters for both protein-coding genes and non-coding RNAs [21]. This systematic analysis enables us to explore the human genome to predict a full list of cell cycle-driving promoters. Our approach is effective in identifying cell cycle genes with low expression levels and is not sensitive to synchronization treatment. Since it is applied at the TSS level, it can distinguish the different isoforms of a gene regulated by alternative promoters. Furthermore, it can also be used to predict cell cycle regulated non-coding RNAs, which we believe will substantially promote our understanding of cell cycle regulation.

## Results

### Transcription factor regulatory scores can discriminate cell cycle from non-cell cycle genes

With the rationale that periodic expression of cell cycle genes is driven by a subset of transcription factors (TFs), we first examined whether cell cycle genes and non-cell cycle genes show different binding strength by TFs. We collected the ChIP-seq data from the ENCODE (The Encyclopedia of DNA Elements) project [20], which provided high-resolution binding events of more than 120 human TFs in multiple cell lines. The binding strength of a TF to the promoters of genes was calculated by a probabilistic model called TIP (Target Identification from Profiles) we proposed previously [22]. This model provides a significantly more accurate measure of TF binding affinity to particular genes than the peak-based method used in many studies [23].

**Figure 2:**
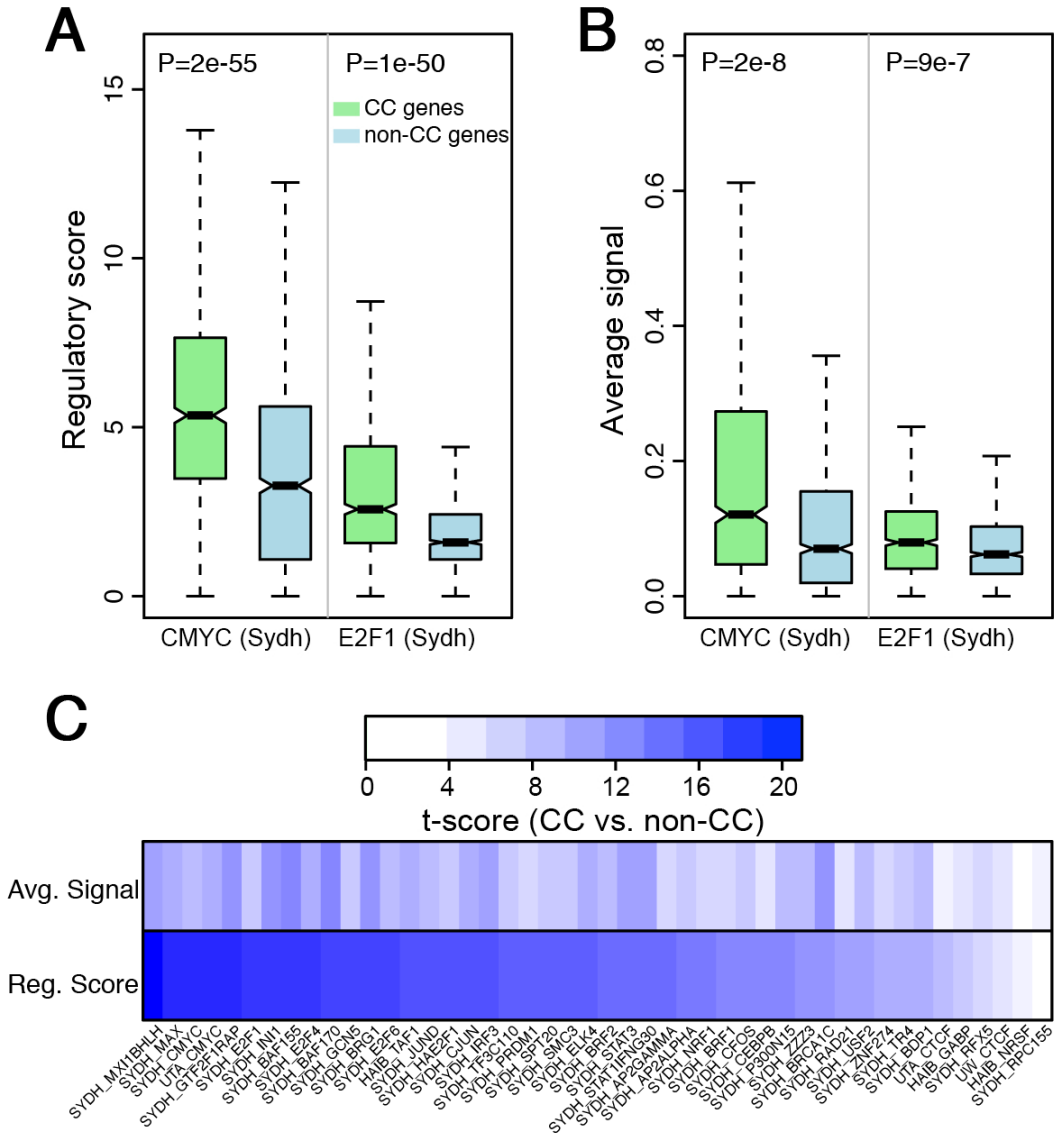
Regulator scores of TFs on genes can discriminate cell cycle (CC) versus non-cell cycle (non-CC) genes. (A) Distributions of regulatory scores for CMYC and E2F1 are significantly different between CC and non-CC genes (P = 2e-55 and P = 1e-50, respectively). (B) The average signals of CMYC and E2F1 show similar distributions between CC and non-CC genes (P = 0.03 and P = 0.05, respectively) (C) The t-scores for CC versus non-CC genes calculated by comparing regulatory scores and average signals of TFs. SYDH, UTA and HAIB are the Lab IDs of a dataset.

We prepared a dataset of cell cycle genes and non-cell cycle genes in HeLa cells that have been experimentally verified with high confidence based on the meta-analysis described in Cyclebase [24]. In the 424 ENCODE ChIP-seq TF binding profiles, 46 were performed in HeLa cells, for which we calculated the regulatory scores using TIP and the average binding signals in a 2 kb DNA region centering at the TSS of all genes (see Methods for details) (Figure 1). Corresponding values of each measurement method were compared between cell cycle genes and non-cell cycle genes using Student's t-test. The regulatory scores for cell cycle genes are significantly contrastive (generally higher than) from those of non-cell cycle genes. As shown in Figure 2A, comparative analysis of the regulatory score distributions of both CMYC and E2F1 show that cell cycle genes tend to have substantially higher regulatory scores than non-cell cycle genes (P = 2e-55 and P = 1e-50, respectively). This is indicative of the significant regulatory roles CMYC and E2F1 have on the expression of cell cycle genes, thus suggesting that they are important features to be used in a cell cycle prediction model [25], [26].

In comparison, the average TF binding signals can also discriminate cell cycle versus non-cell cycle genes, but with much lower significance levels. For example, when average signals of CMYC and E2F1 binding were calculated, we observed less significant difference in values between cell cycle and non-cell cycle genes. The P-values of average signal comparisons are 2e-8 for CMYC and 9e-7 for E2F1 (Figure 2B), indicating that average signals are less effective classifiers for predicting cell cycle genes than regulatory scores.

Other than CMYC and E2F1, many other TFs also reflect significant differences in binding strengths between cell cycle and non-cell cycle genes, especially when TIP is utilized (Figure 2C and Suppl. Table S1). This suggests that the discriminatory efficacy of regulatory scoring is maintained throughout a high percentage of TFs and is not confined to a particular subset of cell cycle regulatory TFs. Thus, we will use regulatory scoring of TF to genes to predict cell cycle genes.

It should be noted that cell cycle genes tend to have higher expression levels than non-cell cycle genes; some of the TF binding difference may reflect the expression level difference rather than their involvement in cell cycle (see Discussion for details). We also note that the cell cycle regulatory function of a TF may not be reflected at the transcriptional level. Among the 46 TFs we investigated, only 6 showed significant periodical expression pattern in cell cycle: E2F1, BRG1, CJUN, RAD21, GABPB and CTCF. The known cell cycle regulators, E2F4 and E2F6, are not significant at the transcriptional level (P>0.01). The model that relates TF binding with cell cycle expression pattern, however, can be used to elucidate the function of TFs in cell cycle regulation by calculating their relative importance.

### Genomic features are predictive of human cell cycle genes

Under the presumption that certain genomic features can discriminate cell cycle genes from non-cell cycle genes, we constructed Random Forest classification models to predict cell cycle genes using ENCODE ChIP-seq-derived TF-binding data and TRANSFAC-derived motif matching data as predictors. More specifically, we calculated the regulatory scores for all human RefSeq genes as described above, resulting in 424 TF binding profiles, each corresponding to a ChIP-seq dataset from the ENCODE project. These binding profiles represent binding strength of TFs to RefSeq genes in a number of different cell lines such as K562, HESC, HeLa, etc. In addition, we also examine the existence of all TRANSFAC TF binding motifs in the promoters of RefSeq genes (from TSS to upstream 1 kb), resulting in a total of 546 motif matching score profiles (see Methods for details). To train the model, we used the cell cycle and non-cell cycle genes identified by microarray experiments in HeLa cells [4]. Consistently, from the 424 TF binding profiles we only included the 46 profiles from HeLa cells in our model.

**Figure 3:**
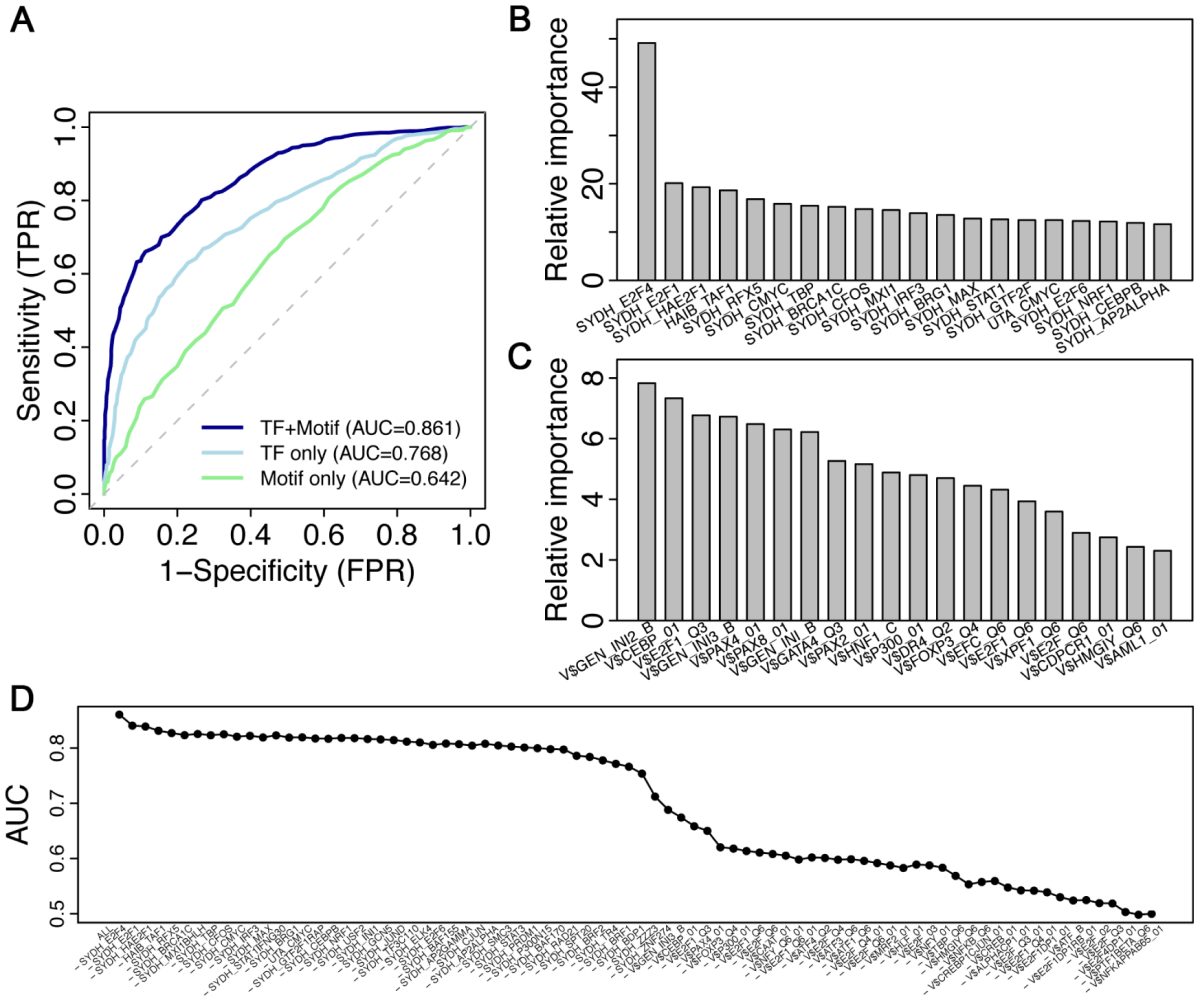
Statistical models for predicting cell cycle genes using Random Forest method. (A) The ROC curves for 3 classification models that use TF-only, motif-only features or a combination of them as predictors. (B) The relative importance (measured as MDG, Mean Decrease in Gini coefficient) of TF features in the combined model (TF+Motif). (C) The relative importance of motif features in the combined model. (D) The change of prediction accuracy (measured as AUC scores) when remove the most important predictor from the full model one by one. Note that cell cycle genes in the training data are from data in Hela cells, and thus we use only TF binding data from the same cell line in our model.

We examined three models for classifying cell cycle versus non-cell cycle genes using Random Forest method. In a TF model, the trans TF-binding features were used as predictors; in a Motif model, the cis motif features are used as predictors; and a TF+motif model uses a combination of all the features. The performance of these models was evaluated by 10-fold cross-validation (see Methods for details). Our results suggest that both TF binding features and motif features are informative for cell cycle gene prediction, with a prediction accuracy AUC = 0.768 achieved by the TF model and AUC = 0.642 achieved by the motif model (Figure 3A). This also suggests that the ChIP-seq derived TF binding features are considerably more predictive than motif features from *in silico* sequence analysis. Strikingly, a combination of both sets of features results in a prediction accuracy that surpasses that of both TF and Motif models, leading to an AUC = 0.861 by the TF+motif model. This indicates that the trans- information captured by ChIP-seq data and the cis- information provided by the motif analysis complement each other during cell cycle prediction.

In a Random Forest model, the contribution of an individual feature to the overall predictive power of the model can be estimated by its relative importance, measured as the Mean Decrease in its Gini Coefficient (MDG) (see Methods for details). Hence, we calculated the relative importance for all TF binding (Figure 3B) and motif features (Figure 3C) in the TF+motif model. Overall, TF features exhibit higher relative importance than motif features, with the best TF feature achieved by SYDH_E2F4 (SYDH is the Lab ID) (Figure 3B) and the best motif feature achieved by V$GEN_INI2_B (Figure 3C). These data confirm that TF-binding regulatory scores are much better predictors than motif matching scores. The high relative importance of E2F4 is consistent with the critical roles it plays in cell cycle regulation [26].

To investigate whether the predictive accuracy of the model is predominantly determined by a few features or by many, we removed features one by one from the model and examine the change in prediction accuracy. In each step we removed the most predictive feature based on their relative importance, then recalculated the accuracy of the new model and re-estimated the relative importance of all remaining features. Our results show that many TF binding features are predictive of cell cycle genes. As shown in Figure 3D, removing the most predictive feature one by one only slowly reduce the AUC score of the model. Such a situation changes until most of the TF binding features have been removed, which leads to a sudden drop in prediction accuracy. At this point, most of the predictors remained in the model are motif features. In fact, we can achieve fairly accurate predictions by selecting a small set of predictors. For instance, when the top 10 TF binding features and the top 10 motif features with highest relative importance in the full TF+Motif model are selected as predictors, we achieve a AUC = 0.850, only slightly lower than the full model (AUC = 0.862).

Apart from the Random forest model, we also implemented other machine learning methods, including support vector machine (SVM) and penalized logistic regression (PLS). Results from all these methods confirm the conclusions from the Random Forest model, e.g. higher predictive accuracy of TF binding features than motif features. Overall, Random Forest gives rise to the best predictive accuracy and thus in this paper we focus on this method in our analysis.

### Cell cycle genes are tissue specific as suggested by predictive models

**Figure 4:**
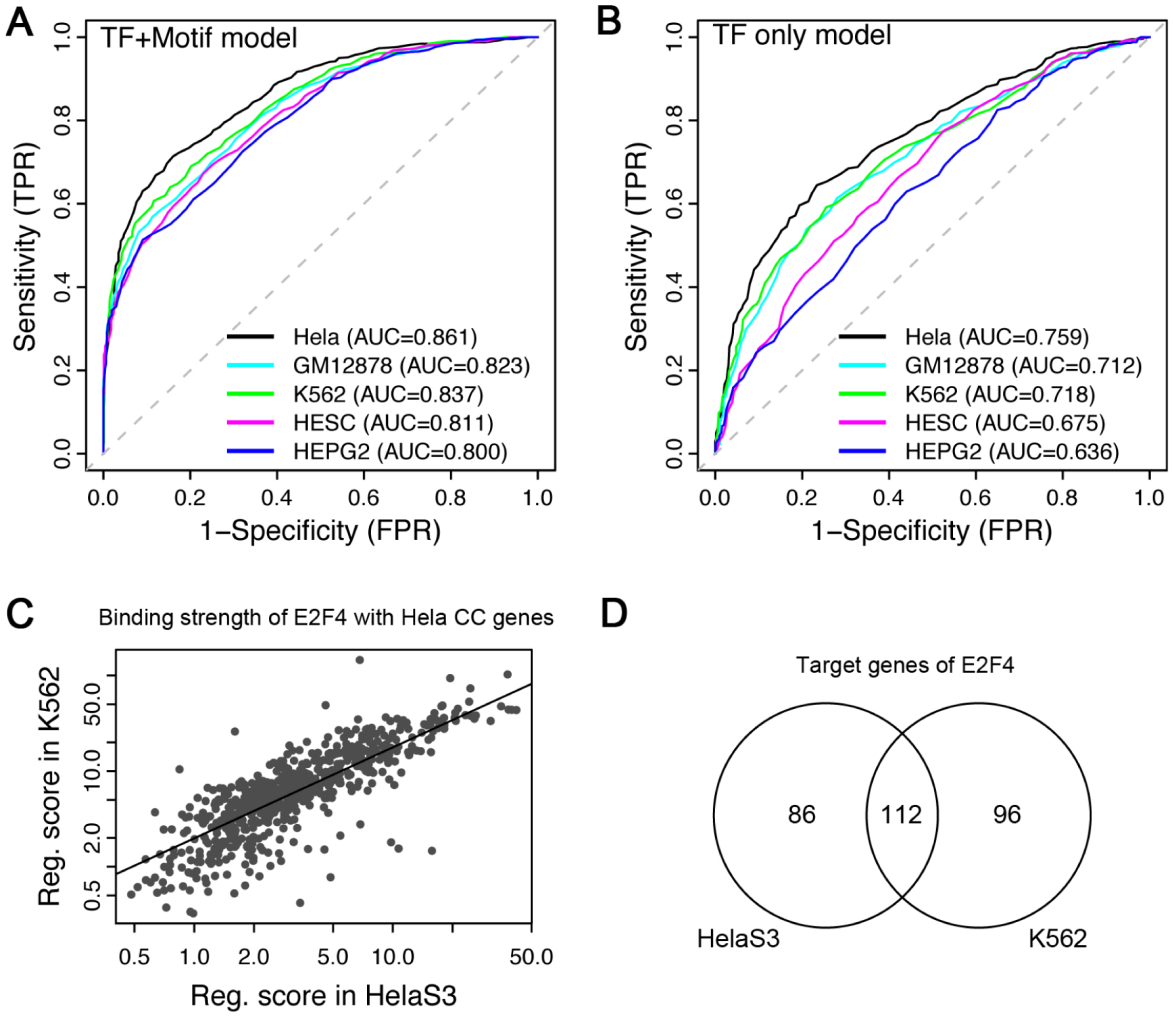
Tissue specificity of cell cycle predictive models. (A) The ROC curves when TF binding data from different cell lines are used as predictors in the combined model. (B) Similar to (A), but results are from TF-only model. (C) The regulatory scores of E2F4 on Hela cell cycle genes in HelaS3 versus K562 cells. Note that a small subset of genes shows strong E2F4 binding only in Hela cells. (D) E2F4 regulates overlapping but different target genes in HelaS3 versus K562. (C) and (D) are based on ENCODE ChIP-seq data.

Since experimentally verified cell cycle and non-cell cycle genes (required to train the model) were determined based on microarray experiments with HeLa cells, we restricted our analysis to HeLa cells in that we only include HeLa TF binding profiles as features in our models. In fact, in the 424 profiles from ENCODE ChIP-seq data, there are 68 from GM12878, 94 from K562, 37 from HESC and 55 from HEPG2 cell lines, respectively (Suppl. Table S2). We thus examined the cell line specificity of our cell cycle gene prediction model. If cell cycle regulation is cell line specific, we would expect to achieve the best prediction accuracy using HeLa TF binding profiles; and otherwise a similar accuracy throughout different cell lines. Our results exhibit highest prediction accuracy when the TF binding features from the HeLa cell line are used for predicting HeLa cell cycle genes, which is the case in both the TF+motif model (Figure 4A) and the TF only model (Figure 4B). The TF sets with ChIP-seq profiles in distinct cell lines contains different TFs. We thereby compared the prediction accuracy of models using the 32 common TFs in HeLa and K562 as predictors. ChIP-seq data from HeLa cells achieve AUC = 0.756 in the TF only model and AUC = 0.860 in TF+Motif model, whereas ChIP-seq data from K562 cells achieves AUC = 0.722 and AUC = 0.831, respectively. These results suggest that at least a subset of cell cycle genes is cell line specific.

Furthermore, we investigate the binding strength of TFs to their target gene promoters in different cell lines. As shown in Figure 4C, the regulatory scores from E2F4 binding to the known cell cycle genes (those used in our model as positive set) are used as a metric to compare differential cell cycle regulation in K562 and HeLa cell lines. Although the scores calculated in HeLa and K562 cells are highly correlated, there is a small set of genes that show differential binding by E2F4, most of which show higher regulatory scores in the HeLa cell line. In addition, when the target genes of E2F4 identified by TIP method in K562 and HeLa are compared, we find that many targets are unique to a single cell line (Figure 4D). This indicates cell line specific binding of TFs to genes and as such, it is not surprising to observe cell line specificity of our cell cycle gene prediction model.

### Prediction of phase specific cell cycle genes

**Figure 5:**
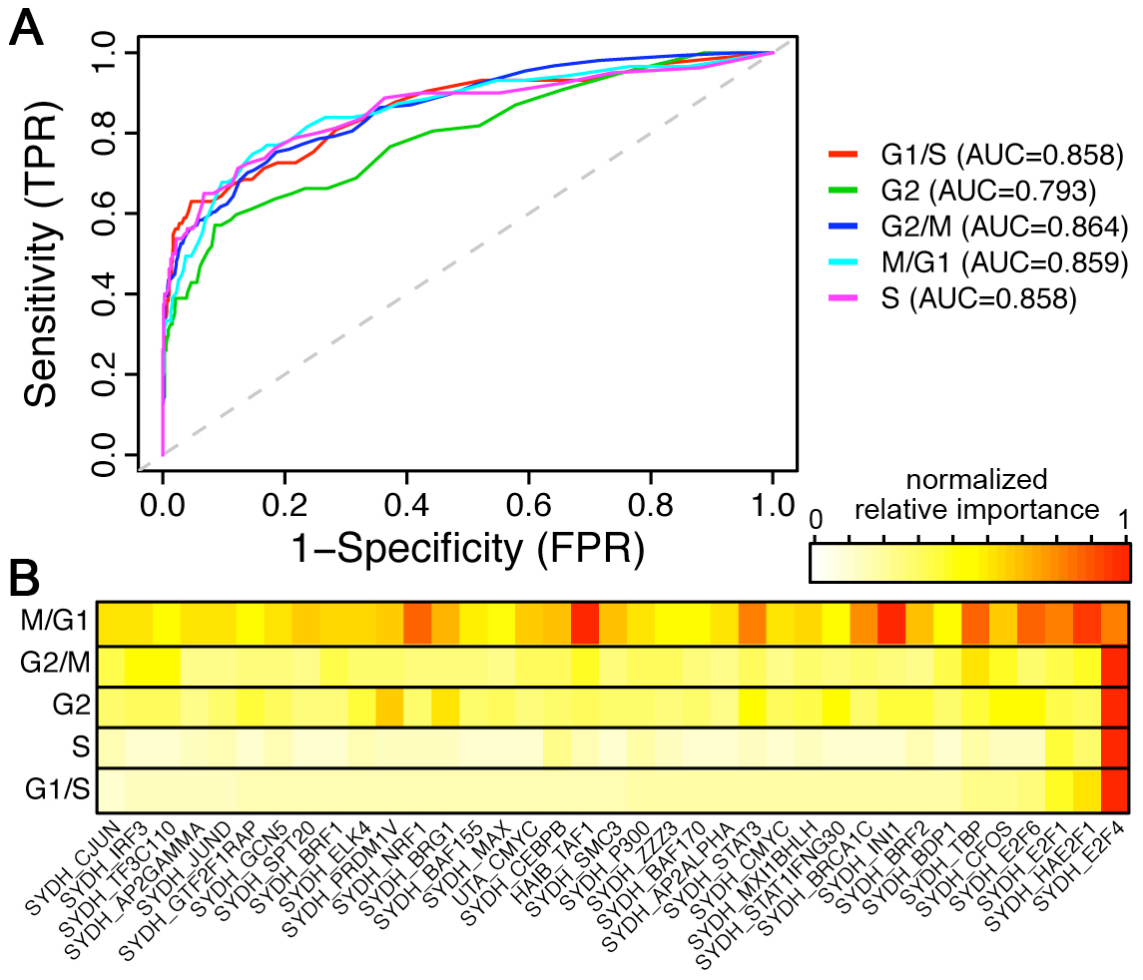
Prediction of phase specific cell cycle genes. (A) ROC curves of models that classify cell cycle genes at specific phase against non-cell cycle genes. (B) The relative importance of different TF features in the 5 phase specific models.

Due to the periodicity of cell cycle genes, genomic features may vary in predictive power across cell cycle phases. Therefore, we examined whether genomic features are phase-specific by applying the Random Forest classifier model to categorize genes expressed in each phase. To generate training data, known cell cycle genes were partitioned into G1/S, G2, G2/M, M, and S categories based on the annotation in Cyclebase [24]. Model accuracy was assessed via 10-fold cross-validation to yield an ROC curve for each cell cycle phase (Figure 5A). Phase-specific cell cycle gene classification via Random Forest proved to be robust as shown by relatively high AUC scores for each phase (Figure 5A). AUC scores of 0.858, 0.793, 0.864, 0.859, and 0.858 were obtained for G1/S, G2, G2/M, M/G1, and S cell cycle phases, respectively.

The normalized relative importance of each genomic feature was calculated to deduce its predictive differentiability in each cell cycle phase (see Methods for details). In all phases, TF features show significantly higher relative importance than motif features. Out of all TF features measured through all cell cycle phases, E2F4 is predominantly the most important predictor in G2/M, G2, S, G1/S phases. However, in the M/G1 phase the prediction accuracy is driven by multiple TF features; interestingly E2F4 still has high relative importance but is not the most predictive feature any more (Figure 5B). In line with these results, we observed that E2F4 targets were enriched in cell cycles genes with peak expression around G2/M and G1/S (Suppl. Figure S2). We note that the ChIP-seq data were performed in unsynchronized cells and reflect TF binding status in a mixed population of cells. We would expect an improvement of phase specific cell cycle gene prediction if phase specific TF binding features were available and utilized as predictors.

### Identification of novel human cell cycle genes

Having shown the effectiveness of our model in predicting cell cycle genes using cross-validation, we applied it to identify new RefSeq genes that are potentially cell cycle regulated. The model was trained and then utilized to predict the cell cycle regulation of a total of 17,023 unclassified RefSeq genes (gene dataset used in model training were excluded). Each gene was assigned a probability indicating the likelihood of a gene to be cell cycle regulated. By setting the threshold to 0.7, we predicted 726 new cell cycle genes with a precision of 92% (positive predictive value, PPV = 0.92). Many of them are subunits of a protein complex that is known to be cell cycle regulated. For instance, Whitfield et al. measured the expression patterns of 12 centromere-associated proteins [27] in HeLa cells, among which 6 were identified as periodically expressed in the cell cycle (CENPA, CENPF, CENPM, CENPL, CENPO, CENPQ and CENPT) [4]. Our analysis predicts 2 additional subunits, CENPK and CENPN, to be cell cycle regulated, suggesting that the model is complementary to microarray based analysis.

To further evaluate the reliability of these predicted cell cycle genes, we carried out Gene Ontology (GO) enrichment analysis on them. The results strongly support cell cycle related functions of these new predicted genes (Suppl. Table S3). As shown, the top enriched GO categories are all cell cycle related, such as chromosome (GO:0005694), cell cycle (GO:0007049), cell cycle process (GO:0022402), cell cycle phase (GO:0022403), M phase (GO:0000279), etc.

Another method of evaluating prediction reliability is to compare them with RNAi knockdown experimental datasets. We downloaded two genome-wide RNAi knockdown datasets published by Mukherji *et al.* [28] and Kittler *et al.* [29], in which cell cycle regulators are identified by knocking down individual genes and examining cell division defects that may result. We find that the novel cell cycle genes we predict tend to exhibit increased likelihood of cell division defect upon RNAi-induced loss-of-function perturbation. In fact, the new cell cycle genes are highly enriched in the cell cycle regulators identified by the two knock-down experiments. A total of 686 and 901 cell cycle regulating genes were identified by Mukherji *et al.* and Kittler *et al.*, respectively, among which 47 were identified by both experiments (P = 4e-4). Out of the 726 novel cell cycle genes we predicted, 50 and 55 were reported to be cell cycle regulating genes by Mukherji *et al.* (P = 2e-4, Fisher's exact test) and by Kittler *et al.* (P = 5e-3, Fisher's exact test) (Figure S1).

**Figure 6:**
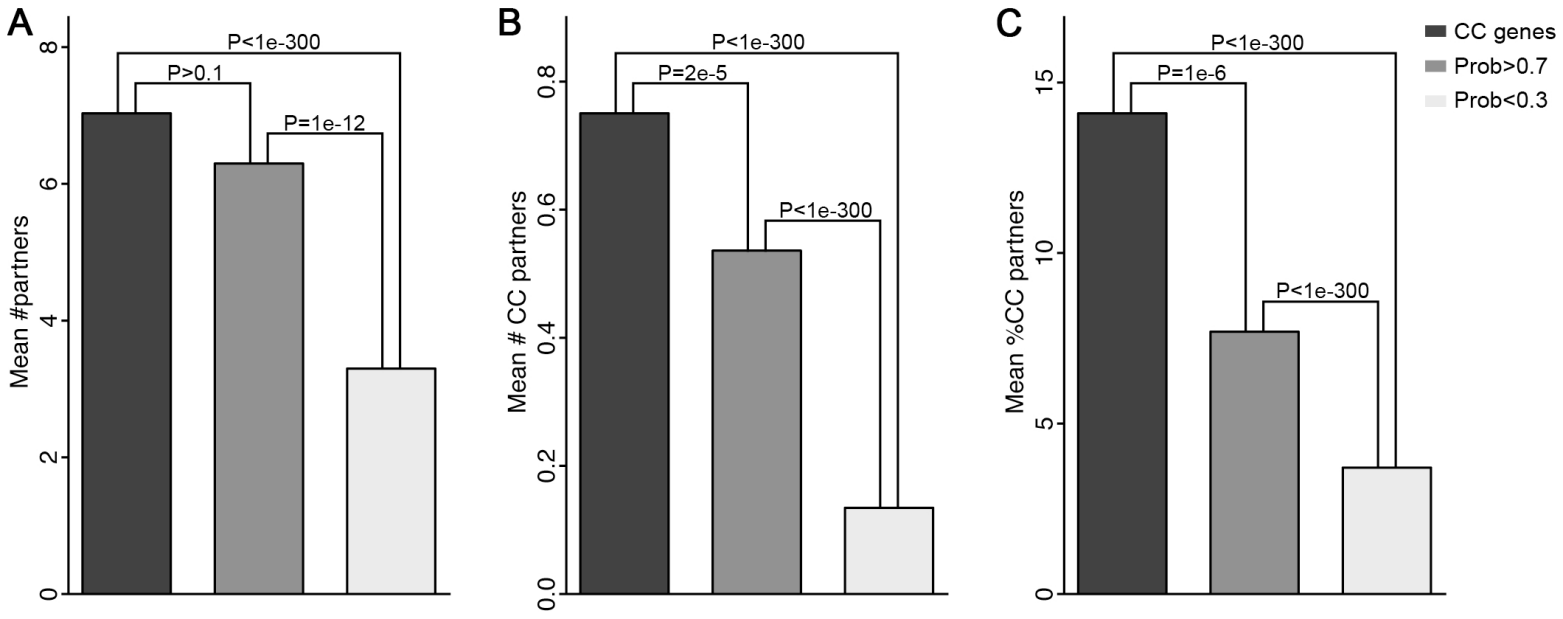
Predicted cell cycle genes are more likely to interact with cell cycle partner in protein-protein interaction network. (A) the average number partners; (B) the average number of cell cycle partners; (C) the average percentage of cell cycle partners. Note all known cell cycle genes are excluded from the predicted cell cycle gene set. The P-values for difference in numbers of partners or cell cycle partners between two gene classes are calculated by Chi-squared test.

Moreover, we examined the interaction partners of known cell cycle genes, the predicted cell cycle genes, and the predicted non-cell cycle genes. We expect that cell cycle genes are more likely to interact with one another and will therefore have more cell cycle partners in the protein-protein interaction (PPI) network. As shown, the known cell cycle genes interact with more partners than other genes (Figure 6A), presumably due to the fact that they are more intensively studied in their interactions, e.g. by yeast two hybrid experiments. Moreover, the known cell cycle genes tend to have more cell cycle partners in terms of both number (Figure 6B) and percentages (Figure 6C). We note that after excluding these known cell cycle genes, the remaining genes used for prediction have substantially fewer partners, cell cycle partners and lower percentage of cell cycle partners. However, compared with predicted non-cell cycle genes (Probability <0.3 in our model), the predicted cell cycle genes (Probability >0.7) interact with significantly more and higher percentage of cell cycle partners (Figure 6B and 6C), implying their functions in cell cycle control.

### Prediction of GENCODE promoters that drive periodical expression

Having shown the effectiveness of our model for predicting cell cycle genes, we then applied it to the GENCODE annotation data, which provides a complete list of human transcripts including protein-coding genes, several categories of non-coding RNAs and so on. For all these transcripts, the precise positions of their TSSs were determined and the expression level associated with each TSS was quantified by CAGE (Cap Analysis of Gene Expression) experiments [30], [31]. We calculated the regulatory scores of these TSSs based on the ENCODE ChIP-seq data and their motif-matching scores for all motifs as we have done for RefSeq promoters (see Methods for details). Finally, the TF+motif Random Forest model trained using the above-mentioned RefSeq cell cycle and non-cell cycle genes was applied to the GENCODE dataset. Thus, by using the regulatory scores and motif-matching scores as features, the model predicts whether a TSS is cell cycle regulated and assigns a probability score to each TSS.

**Figure 7:**
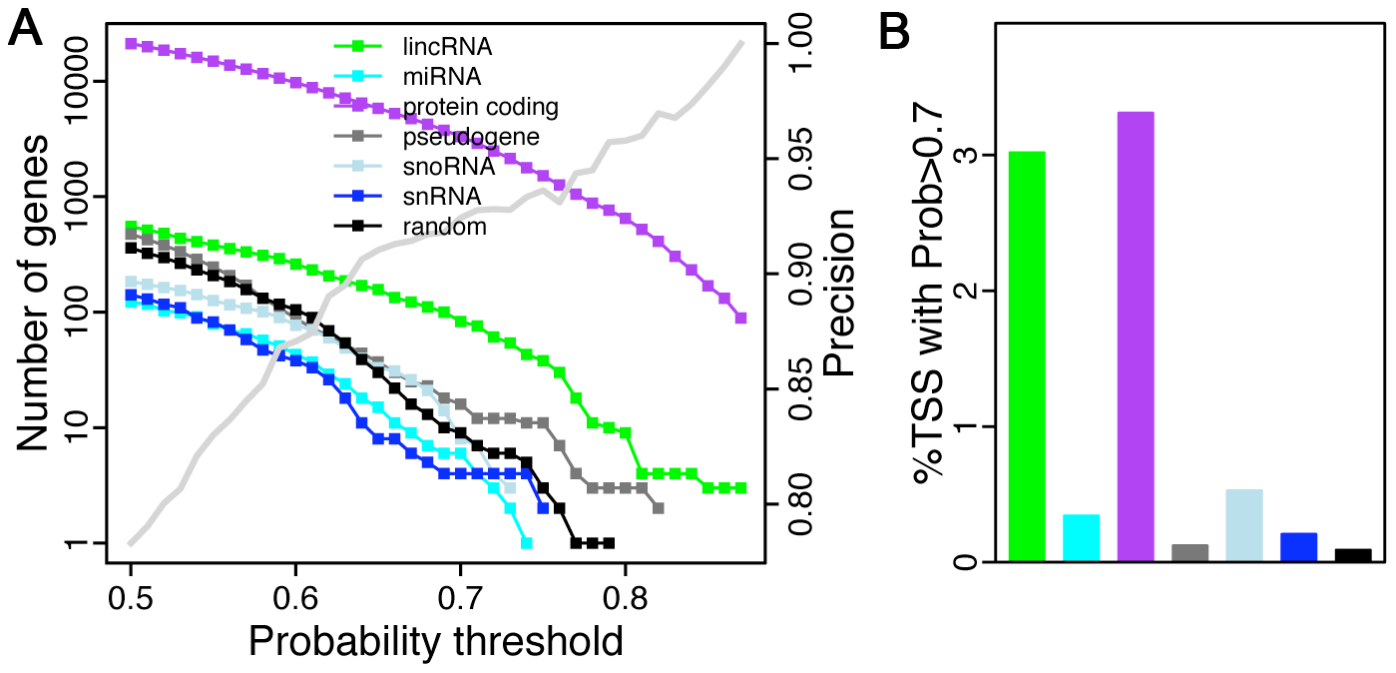
Prediction of cell cycle related promoters. Model is applied to ∼138,000 GENCODE annotated promoters to identify novel cell cycle genes of different types. (A) The number of cell cycle related genes identified the model when different threshold is used. The precision (1-FDR) is shown as the increasing grey line. (B) The percentage of different types of genes that are predicted to be cell cycle related at threshold of 0.7 (Prob>0.7). FDR: false discovery rate.

We predicted the probability of cell cycle regulation for all GENCODE annotated human TSSs using our model. These TSSs are associated with different genomic feature categories including protein-coding genes, microRNAs, lincRNAs, snRNAs, snoRNAs and pseudogenes. As negative controls, we also included 10,013 randomly selected genomic locations (i.e. artificial TSSs) from the genome and predict their probability to be cell cycle regulated using our model. Certainly, the number of positive predictions is determined by the threshold setting and the precision (also called PPV, positive predictive value, the percentage of true positives in all predicted positive predictions) at each threshold can be estimated by cross-validation in our training data (Figure 7A). To have a confident set of predictions, we set up a stringent threshold (Probability score >0.7) in the following analysis, corresponding to a PPV = 0.92. At this threshold, we identify 3,322 protein-coding, 83 lincRNA, 6 miRNA, 8 snoRNA, 4 snRNA, 16 pseudogene, and 9 artificial TSSs that are predicted to be cell cycle regulated (Suppl. Table S4). The percentage of cell cycle regulated TSSs for each genomic feature category is shown in Figure 7B. As shown, the percentage of positive artificial TSSs is very low (<0.1%), indicating a high precision of our predictions. Similarly, the percentage of positive pseudogene TSSs is also very low (1%), since most of them are untranscribed “junk DNA”. But compared to the randomly selected artificial TSSs, it is possible that some pseudogene TSSs are actually active and expressed in cell cycle a dependent manner. Strikingly, lincRNA and protein-coding genes show similar percentage of cell cycle regulated TSSs (∼3%) (Figure 7B), indicating that lincRNAs might also be important in cell cycle regulation. Cell cycle regulated miRNA, snoRNA, and snRNA are identified in relatively low percentages, possibly due to low quality of annotation in their TSSs. For instance, annotation of miRNAs usually begin at the +1 start site of the corresponding pre-miRNA (∼110 bp) as opposed to the genuine TSS of pri-miRNA.

GO enrichment analysis was performed on the predicted cell cycle regulated TSSs associated with GO terms, most of which are for protein-coding genes. The results suggest that these positive predictions are highly enriched in gene categories involved in or related to cell cycle functions (Suppl. Table S5). Almost all of the top enriched GO terms are cell cycle related, e.g. cell cycle (GO:0007049), chromosome (GO:0005694), mitosis (GO:0007067), etc.

Many genes possess multiple transcript isoforms with alternative TSSs and our model can predict the probability of each TSS to be cell cycle regulated. In fact, our results indicate that different isoforms of the same gene may be either cell cycle regulated or not cell cycle regulated, namely have distinct functions with respect to cell cycle regulation. For example, the gene DBF4 (with Ensembl ID ENSG00000006634) is annotated to have 8 different TSSs by GENCODE, which forms two TSS clusters. The first cluster contain 6 TSSs, which are all predicted to be cell cycle regulated with a probability score >0.7; whereas the second cluster (11 kb away from the first cluster) contains 2 TSSs with probability score of 0.296 and 0.190 respectively. The DBF4 protein is known to be essential for initiation of DNA replication [32] and the transcription of its promoter is activated through cell-cycle box (MCB) transcription elements [33]. Assuming the TSS annotation is correct, our analysis imply that only the first cluster of transcript isoforms are regulated in a cell cycle dependent manner; and that the two isoforms in the second cluster may not be periodically expressed during the cell cycle, either not being involved in cell cycle regulation or impacting cell cycle in a different way from the first cluster of isoforms.

## Discussion

Compared to the average binding signals of TFs in promoters, the regulatory scores we define are more informative for predicting cell cycle genes (Figure 1). Regulatory scores can be regarded as weighted average binding signals of TFs around the TSS of genes. For each TF, a specific weight is assigned to each nucleotide position in the 10 kb DNA region centering at TSS based on the characteristic binding profile of the TF. Thus regulatory scores can more accurately capture the regulatory potential of a TF to genes than average signals. When utilized as predictors for classifying cell cycle versus non-cell cycle genes, they generally reveal greater differentiability between the two gene classes, suggesting they are more powerful classifiers. In fact, the Random Forest model that utilizes average signals for the same set of TFs as predictors achieves a classification accuracy AUC = 0.683, which is similar to the accuracy of the motif only model (AUC = 0.642), and is significantly lower than the TF only model that is based on regulatory scores (AUC = 0.768). Thus, it seems that by combining with machine-learning methods, the regulatory scores calculated from ChIP-seq data might also be promising in other applications, for example, predicting tissue specificity or conditionally expressed genes.

Our analysis indicates that cell cycle regulation in different cell lines may not be exactly the same but shows certain cell line specificity: cell cycle genes identified in Hela cells can be best predicted by ChIP-seq TF binding profiles from the same cell line. Although the binding strengths of E2F4 to HeLa cell cycle gene promoters in both HeLa and K562 cell lines are comparable, there exists an observable small subset of genes exhibiting highly differential E2F4 binding affinities; with the majority of them showing more vigorous binding in HeLa cells. (Figure 4C). In fact, a large percentage of E2F4 target genes identified by ChIP-seq experiment are HeLa or K562 specific (Figure 4C). Moreover, we compared the cell cycle genes identified via cDNA microarray experiment in HeLa cells by Whitfield *et al.* (588 genes) [4] and in fibroblast cells by Iyer et al. (480 genes) [12], and discover that only 155 are cell cycle genes in both cell lines. Contrastingly, TF binding data from other cell lines also prove predictive to HeLa cell cycle genes with reasonably high accuracy, indicating somehow a similar language of cell cycle regulation between cell lines. From these observations, it seems that to some extent, cell cycle regulation is cell line specific yet there may exist a core set of genes that are cell cycle regulated across all cell lines.

The relative importance of predictors in our model suggests that E2F4 is essential in cell cycle gene regulation. In addition, the TF binding profiles for E2F1 and E2F6 also show significantly differential binding strengths between cell cycle and non-cell cycle genes, and exhibit high relative importance in our model. These results are in accordance with existing literature, which assert that the E2F family of transcription factors plays an inextricable role in driving and regulating cell cycle [34]. E2Fs are regulated by the pRB-family and pRB-related proteins (e.g. p130 and p107), that are inactivated upon CDK-mediated phosphorylation [35]. Additionally, E2F exhibit dual properties in that E2F1–E2F3 act as activators and E2F4–E2F8 as repressors [36]. In particular, E2F4 is shown by ChIP-chip analysis to have a plethora of gene targets involved in every phase of the cell cycle [26], [34], [37]. Principal targets of E2F4 include genes involved in cell cycle regulation, DNA replication, DNA repair, chromatin remodeling, and cell cycle checkpoints [26]. Evidently, these cellular processes are all associated with cell cycle genes thereby forming an integrated network of gene regulation [26]. Because E2F4 is a negative regulator, it must be constantly repressed by pRb and only expressed intermittently to allow the cell cycle to progress. This allows cellular processes controlled by E2F4 to occur in a phase-specific fashion (i.e. DNA repair during S/G2, DNA replication during S, and chromosome remodeling during G2/M) [26]. Additionally, a comparative genomics study carried out by Linhart *et al.* proposes that there is substantial decrease in E2F4 binding during M/G1 phase of the cell cycle [38]. This is in accordance with our results which show a decrease in normalized relative importance of E2F4 during the M/G1 phase (Figure 5B). Overall, these results suggest that E2F4 is repressed upon termination of mitosis and subsequently de-repressed upon initiation of G1 in daughter cells. The fact that E2F4 binding is an effective discriminatory cell cycle-associated TF binding feature demonstrates that our prediction model is indeed capable of utilizing key inherent cellular predictors to classify a wide variety of genes.

Previous studies have shown that TF binding signals are predictive to the expression level of genes, accounting for >60% variation of gene expression [39]–[42]. Here we show that TF binding data can be used to predict cell cycle genes. The predictive power of regulatory scores which capture the trans- information of genes, can be further improved by the cis- information of these genes, or the motif matching scores in their promoters. Our model which uses TF+motif predictors achieves a classification accuracy of AUC = 0.861, suggesting the regulatory code for cell cycle genes is largely harbored in their promoter regions. The TF binding data and the motif information complement each other, because (1) none of the two data are complete (e.g. the ChIP-seq data of many critical cell cycle regulatory TFs are not available) and (2) the trans- TF binding data from ChIP-seq captures regulatory information not only at the transcriptional level but also at the epigenomic level, since TF binding is significantly affected by epigenomic modifications (e.g. histone modifications and DNA methylation). The periodical expression pattern of cell cycle genes is also regulated at the post-transcriptional level, e.g. by miRNAs, and we believe that predictive accuracy of our model can be further improved when such information are included. One caveat of the model is that ChIP-seq experiment captures TF binding in a population of unsynchronized cells, which limits our model from more precisely elucidating the cell line specific and phase-specific regulation of TFs.

As we have described, microarray-based methods are less effective in identifying cell cycle genes expressed at low levels. For this reason, cell cycle genes detected from microarray experiment tend to have higher expression levels compared to those of non-cell cycle genes. In fact, when we statistically compare the expression levels of cell cycle genes versus non-cell cycle genes in HeLa cells, we observe significant expression disparity (P = 3e-42, Wilcoxon rank sum test). Furthermore, it has been demonstrated previously that TF binding is predictive of gene expression levels [39]–[42], which makes expression level a confounding factor to account for when classifying cell cycle versus non-cell cycle genes: the model we propose here may be restricted to prediction of high versus low gene expression rather than cell cycle genes. This also explains why most of the TFs show very differential regulatory scores between cell cycle and non-cell cycle genes (Figure 2), but only 6 of them show periodical expression pattern during cell cycle in HeLa cells. To address this confounding issue, we prepare a set of non-cell cycle genes that have similar expression levels with cell cycle genes in HeLa. When the regulatory scores are compared between these genes and cell cycle genes, fewer TF features show significant difference but the key cell cycle regulators (e.g. E2F1 and E2F4) still maintain significant difference. This suggests that these key regulators do in fact, bind differentially with cell cycle versus non-cell cycle genes even after decoupling them from the influence of expression levels. If average signals of TF features are compared, none of the TFs show differential binding at the 0.001 significance level, again demonstrating the advantage of regulatory scores. More importantly, when these expression-matched non-cell cycle genes are used as the negative training set, we can still accurately predict cell cycle genes with AUC = 0.706 using the TF only model and AUC = 0.814 using the TF+motif model. Thus, we can conclude that the model is effective for cell cycle gene prediction when the influence of expression level is eliminated and a very conservative training set is used.

Most previous cell cycle research is focused on protein-coding genes, while in-depth systematic investigation of cell cycle non-coding RNAs have not been conducted. A recent paper examined the promoters of 56 cell-cycle genes using tiling array and revealed extensive non-coding transcription near these genes [43]. This explorative study highlights the potential importance of regulation by non-coding RNA during cell cycle division. We applied our model to more than 130,000 human TSSs annotated by GENCODE project and systematically predicted the probability of these TSSs to act as cell cycle driving promoters. The GENCODE TSS list contains TSSs for not only protein-coding genes but also for several classes of non-coding RNAs such as miRNAs, lincRNAs, snoRNAs, and snRNAs. Our predictions suggest that there is at least equal percentage of lincRNAs that are cell cycle regulated as there are protein-coding genes. Further experimental investigation of these non-coding RNAs should provide further insight into the non-coding world of cell cycle regulation.

The enormous amount of genomic data from the ENCODE project provide valuable resources for biological studies. However, how to more efficiently make use of such data to facilitate hypothesis driven studies is still an open question. Here we show an example that combines large-scale ChIP-seq data from ENCODE with motif data from genome sequence analysis and cell cycle microarray data from small-scale laboratory studies. The framework introduced in this paper may also be applied to address other biological questions such as identifying tissue specific expression of genes, gene classes, and environment-induced gene expression and so on.

## Methods

### Microarray cell cycle time course data

In this work, we used a supervised model to predict human cell cycle genes. To train the model, we obtained the known cell cycle genes and non-cell cycle genes from the data produced by Whitfield et al [4], which measured gene expression during the cell division cycle in HeLa cells using microarray experiments. The data contain four different cell cycle time course series, each providing a list of cell cycle genes. To have a confident cell cycle gene list, we referred to the meta-analysis performed by Cyclebase [24], which combined the results of all these four time courses. The cell cycle genes (positive training set) were selected as those with a significant combined P-value for periodicity (P<0.011), while the non-cell cycle genes (negative training set) were selected as those that were not significant in any of the four time courses (P>0.1). In total, we obtained 853 cell cycle Refseq genes and 1051 non-cell cycle Refseq genes. The phase-specificity of cell cycle genes were determined based on their peak expression time provide by Cyclebase. In Cyclebase, each cell cycle genes is assigned a value of 0–100 indicating their peak expression time with G1 (0–47), S (47–70), G2 (70–90) and M (90–100). Accordingly, we selected 138 M/G1 (95–100 or 0–20), 257 G2/M (80–95), 253 G2 (70–90), 175 S (47–70) and 185 G1/S (20–60) specific Refseq genes for model training.

### Calculation of transcription factor regulatory score

We calculated the binding affinity of transcription factors to the promoter of a gene based on their corresponding ChIP-seq data. The ChIP-seq data provides the binding signal of a TF at each nucleotide of the genome. We utilized the method called TIP (Target Identification from Profile) to quantify the regulatory relationships between TFs and target genes [22]. Given the ChIP-seq data of a TF, TIP builds a characteristic, averaged profile of binding around the TSS of all genes and then uses this to weight the sites associated with a given gene, providing a ‘regulatory’ score of this for each gene. From the ENCODE project [20], we downloaded a total of 424 ChIP-seq data, representing the binding data for about 120 different TFs in more than 10 cell lines such as HelaS3, HESC, K562, etc. For each of them, we calculated the regulatory scores for all RefSeq genes, giving rise to a matrix of 34,299 (RefSeq genes) rows and 424 columns (ChIP-seq datasets).

The average binding signals of a TF with a gene is calculated by averaging the ChIP-seq signal of all nucleotide position in the promoter DNA region (a 2 kb DNA region centering at the TSS) of the gene.

### Calculation of motif matching scores in promoter of genes

We downloaded 565 vertebrate motifs from the TRANSFAC database [44], which represent the potential binding sites of DNA binding proteins, mostly transcription factors. We also downloaded the promoter sequences (from TSS to upstream 1000 bp of a gene) of 34,229 human RefSeq genes from the UCSC Genome Browser [45]. For each promoter sequence, we used the MATCH program [46] to examine the presence of these TF binding motifs. The pre-calculated cut-offs provided by MATCH were used to minimize the false positive rate. The MATCH program provides all the potential binding sites and their matching-scores of all of the RefSeq gene promoters. Based on these outputs, we constructed a binding score matrix [B_i,j] of size N×M, in which each row representing a RefSeq gene (N = 34,229) and each column corresponding to a motif (M = 565). Each element B_ij was calculated by aggregating the matching-scores of all the binding sites of the motif j in the promoter of the gene i. The score was set to 0 if there is no binding site in the promoter of a gene.

### Predicting cell cycle genes using Random Forest

The Random Forest ensemble classifier was used to as a machine-learning model to predict genes as cell cycle or non-cell cycle. A prepared dataset containing known cell cycle genes (annotation derived from RefSeq and Cyclebase) and their associated TF features derived from ENCODE and TRANSFAC databases was used to train the model. This dataset contained 863 known cell cycle genes and 1051 known non-cell cycle genes. To generate the final training dataset, 81 TFs were chosen as pre-selected features resulting in a total of 69,903 cell cycle TF-gene pairs and 85,131 non-cell cycle gene pairs. Each cell cycle gene was assigned a positive binary value (1) and each non-cell cycle gene was assigned a negative binary value (0). Model accuracy was assessed using 10-fold cross-validation in the following procedure. First, each fold was carried out by randomly dividing the training dataset into 10 partitions, irrespective of binary assignment. Second, 9 partitions were used to train the Random Forest model and the remaining partition was used as a test set to determine model performance. This is repeated 9 more times to yield the averaged sensitivity ([#True Positives]/[#True Positives+#False Negatives]) and specificity ([#True Negatives]/[#True Negatives+#False Positives]) of the model. This allows construction of a Receiver Operating Characteristic (ROC) curve, which is a direct representation of the relationship between sensitivity and specificity. The area under the ROC curve (AUC) is calculated via Riemann summation of 100 trapezoidal partitions. Calculation of the AUC is the main evaluator of Random Forest model accuracy in this study; AUC = 1 corresponds to 100% model accuracy and AUC = 0.5 corresponds to random classification by the model, thus completely non-discriminatory.

The relative importance (RI) of a predictor in a Random Forest model can be measured by the metric “%IncMSE” (increase of mean squared error) [47]. Given a trained model, “%IncMSE” measures the increase of prediction error in the test data when the values for each individual predictor are permuted. The permutation of an important predictor will be expected to lead to a considerable prediction error increase and therefore a large “%IncMSE”. The relative importance of each genomic feature is model specific. The relative importance of features for predicting phase-specific cell cycle genes is calculated respectively from the corresponding phase-specific model.

The R package “randomForest” was utilized to implement these models.

### Predicting periodical expression driving GENCODE promoters

Transcription start sites (TSS) information was derived from the GENCODE gene annotation project [30] and the high confidence TSS sets from GENCODE version 7 was used. This set includes a total of 137,874 TSSs for different gene categories, including protein coding genes (100,417), miRNAs (1,755), lincRNAs (2,751), pseudogenes (13,164), etc. In the dataset, many genes are associated with multiple TSSs which corresponds to these genes having alternative promoters. As we have done for the RefSeq genes, we calculated the TF regulatory scores and the motif matching scores for all these TSSs using the above-described methods.

We trained Random Forest using the RefSeq gene training data described in the preceding section, and then apply the model to predict the probability of these TSSs function as cell cycle driving promoters. Meanwhile, we generated ∼10,000 random TSSs that are evenly distributed in the genome and fed them into the model as controls. Given a cut-off, the false discovery rate (FDR) of our model can be estimated by calculating the ratio of F_rand to F_real, where F_rand and F_real are the fractions of predicted cell cycle driving random TSSs and real TSSs (i.e. TSSs with probability above the cut-off).

### Enrichment analysis of E2F4 targets in cell cycle

We investigated the distribution of transcription factor target genes in the cell cycle. First, we sorted the cell cycle genes in HeLa cells according to their peak expression times. Then we examined the enrichment of the target genes of a given transcription factor in each sliding window of the cell cycle. We used a window size of 30 degrees with 10 degrees overlapping between neighboring windows. We used the Fisher's exact test to determine the significance of enrichment of target genes for a transcription factor in each cell cycle window (Suppl. Figure S2).

### Other datasets and bioinformatic analysis

Systematic gene knockdown data for cell division genes screening are available from Mukherji *et al.* [28] and Kittler *et al.* [29]. In the two studies, the majority of human protein-coding genes were knocked down in U2OS and HeLa cells, respectively, to identify cell cycle regulating genes. We examined and calculated the significance the enrichment of our predicted cell cycle genes in gene sets identified by Mukherji et al. and Kittler et al. using Fisher's Exact test (Suppl. Figure S1).

To examine the enrichment of genes of different gene ontology (GO) categories in our predicted cell cycle gene set, we performed GO enrichment analysis by using the web-based tool from DAVID database (the Database for Annotation, Visualization and Integrated Discovery), which calculated significance of enrichment based on Fisher's exact test [48]. In the analysis, we removed the cell cycle and non-cell cycle genes in the training set to avoid their impact and limit bias

The human protein-protein interaction data is downloaded from the Human Protein Reference Database (HPRD, Release 8) [49]. Human RefSeq gene annotations are obtained from the UCSC Genome Browser database [45].

## Supporting Information

Figure S1 Validation of novel predicted cell cycle genes from large-scale gene knockdown experiments. (A) Comparison of predicated cell cycle genes with knockdown results from Mukherji et al. (B) Comparison of predicated cell cycle genes with knockdown results from Kittler et al. (C) Comparison of knockdown results between Mukherji et al. and Kittler et al.
(TIF)

Figure S2 Enrichment of E2F4 target genes during the cell cycle. Human cell cycle genes are ordered based on their peak expression time in the cell cycle, and enrichment of E2F4 targets in each time window is calculated by using Fisher's Exact test.
(TIF)

Table S1 Regulatory scores and average signals of transcription factors for cell cycle and non-cell cycle genes.
(XLS)

Table S2 The list of 424 ENCODE ChIP-seq experiment for transcription factors.
(XLS)

Table S3 Gene Ontology analysis results of novel predicted cell cycle Refseq genes.
(XLS)

Table S4 The number of positive prediction of different classes of GENCODE annotated genes at different thresholds. PPV (Positive Prediction Value) indicates the prediction precision estimated by cross-validation.
(XLS)

Table S5 Gene Ontology analysis results of GENCODE annotated protein-coding genes that are predicated to be cell cycle regulated.
(XLS)
