# Supplementary figures and images for: Transcription Factor Binding Profiles Reveal Cyclic Expression of Human Protein-coding Genes and Non-coding RNAs
Source: PLoS Comput Biol. 2013 Jul 11;9(7):e1003132. doi: 10.1371/journal.pcbi.1003132 (PMC3708869; doi:10.1371/journal.pcbi.1003132)

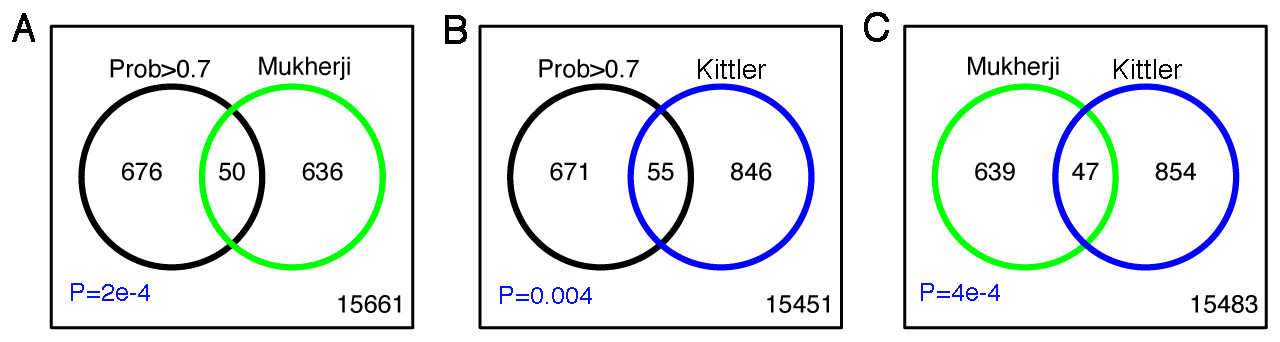

Supplement: Figure S1 — Validation of novel predicted cell cycle genes from large-scale gene knockdown experiments. (A) Comparison of predicated cell cycle genes with knockdown results from Mukherji et al. (B) Comparison of predicated cell cycle genes with knockdown results from Kittler et al. (C) Comparison of knockdown results between Mukherji et al. and Kittler et al. (TIF) [file pcbi.1003132.s001.tif]

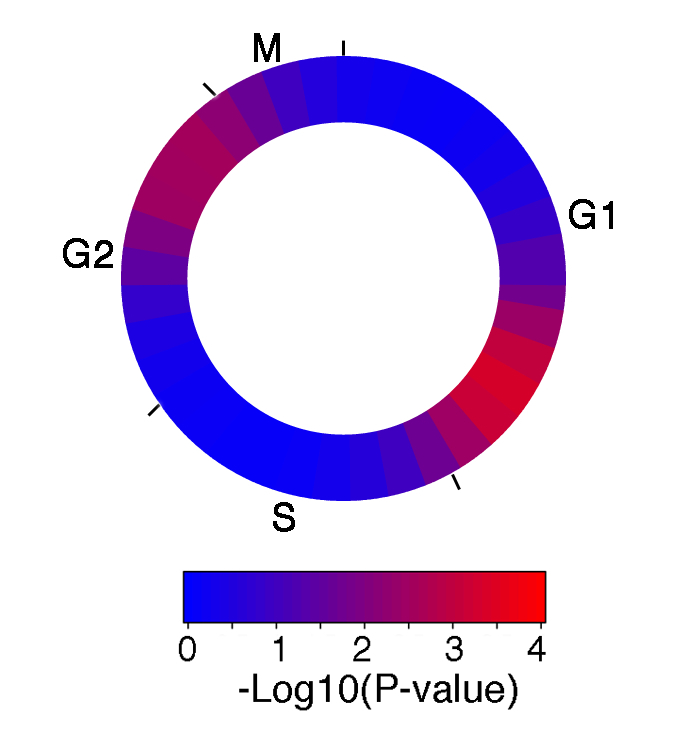

Supplement: Figure S2 — Enrichment of E2F4 target genes during the cell cycle. Human cell cycle genes are ordered based on their peak expression time in the cell cycle, and enrichment of E2F4 targets in each time window is calculated by using Fisher's Exact test. (TIF) [file pcbi.1003132.s002.tif]
